# Mendelian Randomization and Bioinformatics Analysis Reveal the Potential Protective Role of Metformin in Primary Liver Cancer

**DOI:** 10.1002/fsn3.71156

**Published:** 2025-11-02

**Authors:** Yongxin Ma, Jiaojiao Qi, Zhiqiang Chen, Yubo Zhang, Kejun Liu, Jiaxin Suo, Bendong Chen, Yang Bu

**Affiliations:** ^1^ Department of Hepatobiliary Surgery General Hospital of Ningxia Medical University Yinchuan Ningxia China; ^2^ Ningxia Medical University Yinchuan Ningxia China; ^3^ Department of Obstetrics Function Center Inspection General Hospital of Ningxia Medical University Yinchuan Ningxia China

**Keywords:** bioinformatics, hepatocellular carcinoma, mendelian randomization, metformin, primary liver cancer

## Abstract

Primary liver cancer (PLC) and metformin are not well understood to be associated. We conducted a Mendelian randomization (MR) analysis using genetic data from IEU OpenGWAS and FinnGen R10, with metformin as the exposure and PLC as the outcome. The inverse variance weighting (IVW) method was the primary analytical approach, with heterogeneity assessed by Cochran's Q test, pleiotropy by MR‐Egger intercept, and outliers by MR‐PRESSO. Bioinformatics analyses further explored potential mechanisms, including differential gene expression, protein–protein interactions (PPI), Gene Ontology (GO) and Kyoto Encyclopedia of Genes and Genomes (KEGG) enrichment analyses, immune cell infiltration analysis, and drug sensitivity analysis. MR results demonstrated a significant association between metformin use and reduced risk of PLC (*β* = −5.6046, OR = 0.0037, *p* = 0.026), with a Benjamini‐Hochberg false discovery rate (FDR) adjusted *p* value of 0.13. However, no causal effect was observed for hepatocellular carcinoma (HCC) or intrahepatic cholangiocarcinoma (ICC). By cross‐referencing transcriptome data from the GEO database GSE241466 with metformin‐related gene loci, 34 overlapping genes were identified. Differentially expressed genes (DEGs) were filtered using |log2FC| > 0 and *p* < 0.05, with five hub genes (DDX52, KIF11, GCDH, MRPL45, and TICRR) being particularly prominent. Functional enrichment analysis revealed involvement in cGMP‐PKG signaling and fatty acid metabolism pathways. Further validation with GEPIA2, TIMER, and TISCH showed correlations between these genes and immune infiltration, while GSCA‐based drug sensitivity analysis suggested therapeutic relevance. In summary, these findings indicate that metformin may reduce PLC risk by modulating metabolic and immune‐related pathways, supporting its potential value as an adjunct therapeutic agent. However, further validation through large‐scale clinical and basic research is warranted.

## Graphical abstract

Using two‐sample Mendelian randomization and bioinformatics, this study found metformin associated with reduced primary liver cancer (PLC) risk (*p* = 0.026), without links to HCC/ICC. Key genes (e.g., DDX52, KIF11) and pathways (cGMP‐PKG signaling, fatty acid metabolism) were identified. Results suggest metformin's protective role in PLC via metabolic and tumor‐suppressive mechanisms, warranting further investigation as a potential therapy.

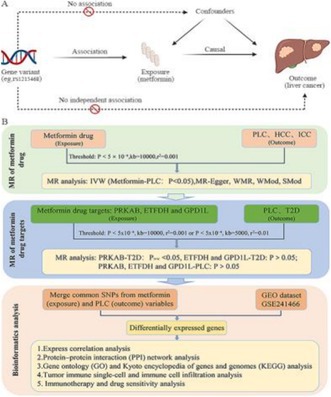

## 1 Introduction

Primary liver cancer (PLC), a malignant tumor developing from hepatocytes or intrahepatic bile duct epithelium, primarily includes hepatocellular carcinoma (HCC), intrahepatic cholangiocarcinoma (ICC), and mixed subtypes (Gao et al. 2020), with approximately 906,000 new cases and 830,000 deaths reported worldwide in 2020 (Sung et al. 2021), ranking as the sixth most prevalent neoplasm and the fourth leading cause of cancer mortality worldwide (Sun et al. 2023). PLC progresses asymptomatically in early phases and acquires metastatic capabilities during progression, often resulting in delayed diagnosis at advanced stages with an unfavorable prognosis (Chidambaranathan‐Reghupaty et al. 2021). Despite considerable progress in therapeutic strategies for PLC—such as surgical resection, radiofrequency ablation (RFA), transcatheter arterial chemoembolization (TACE), targeted therapy, immunotherapy, and even liver transplantation—the prognosis remains poor. Elevated recurrence and metastasis rates compared with other malignancies have hindered substantial progress in 5‐year overall survival (Siegel et al. 2022; Torimura and Iwamoto 2022). While the etiology of PLC is not fully elucidated, epidemiological investigations have established key risk factors—notably hepatitis B (HBV) and C (HCV) infections, along with chronicnecro‐inflammatory hepatic injury from alcohol or non‐alcoholic fatty liver disease (Li et al. 2021; Papadimitriou et al. 2021). Therefore, it is crucial to further investigate the potential risk or protective factors, pathogenesis, and potential novel adjuvant therapies for PLC.

Metformin, a first‐line therapy for type 2 diabetes (T2D), has attracted growing attention due to its potential anti‐tumorigenic properties (Mallik and Chowdhury 2018). Although numerous clinical studies have investigated its efficacy across various cancers, inconsistent findings have precluded definitive conclusions (Barakat et al. 2022; Bragagnoli et al. 2021). Chen et al. (2023) demonstrated that metformin may reduce both the incidence and mortality of multiple malignancies, including breast, hepatic, and pancreatic cancers. Its antitumor effects involve complex interrelated metabolic and signaling pathways, including: (1) Insulin‐like growth factor‐1 (IGF‐1) inhibition; (2) Mitochondrial electron transport chain suppression reducing ATP production, thereby activating AMP‐activated protein kinase (AMPK); (3) Mammalian target of rapamycin complex 1 (mTORC1) blockade; (4) Nuclear factor‐κB (NF‐κB) inactivation; (5) Cancer cell oxidative phosphorylation impairment (Li et al. 2020; Xue et al. 2021). Current evidence on the relationship between metformin and PLC remains limited and inconclusive, with causality yet to be established. Exploring potential causal links is essential for identifying the disease's predisposing factors and informing clinical treatment strategies.

Although randomized controlled trials (RCTs) represent the gold standard for establishing disease‐intervention causality, their implementation is often prohibitively expensive and time‐intensive. Given these practical constraints in investigating the metformin‐PLC relationship, Mendelian randomization (MR) analysis provides an efficient alternative approach. MR employs genetic variants as instrumental variables (IVs) to assess exposure effects, mitigating confounding and facilitating robust causal inference when core statistical assumptions are met (Richmond and Davey Smith 2022). It has been extensively utilized to clarify associations between exposures and outcomes, offering a significant complement to observational studies (Yuan et al. 2022). Currently, no research has examined the causal links among metformin, its targets, and PLC pathogenesis. Using MR, this study investigates metformin's causal effects on PLC development. Given HCC represents most PLC cases, we further employed bioinformatics to systematically elucidate metformin's potential mechanisms in HCC progression, thereby informing therapeutic strategies.

## 2 Materials and Methods

### 2.1 Study Design

**FIGURE 1:**
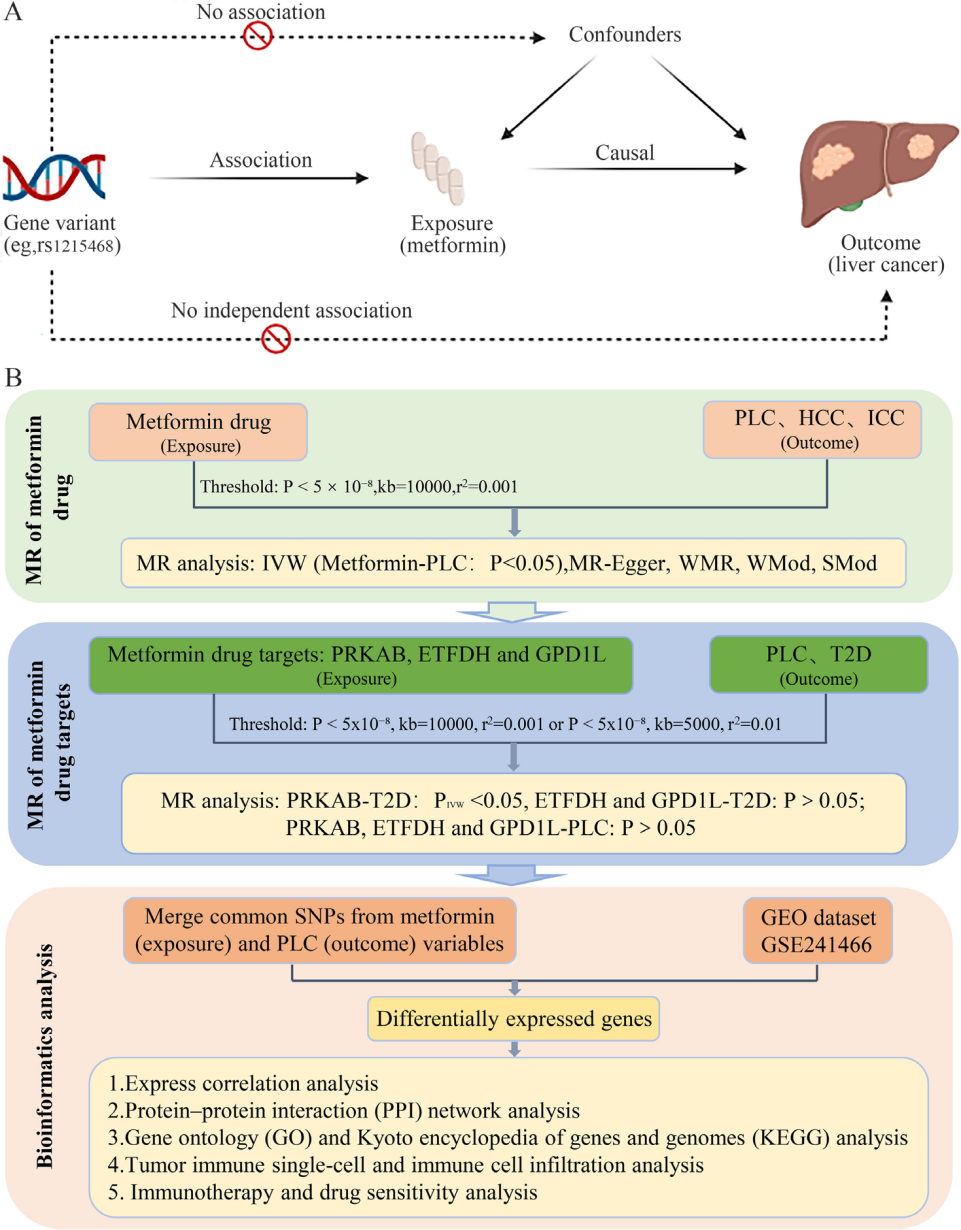
Description of the study design in this MR study. (A) MR analyses depend on three core assumptions. (B) Flowchart of the present study. PLC, primary liver cancer; HCC, hepatocellular carcinoma; ICC, intrahepatic cholangiocarcinoma; LD, linkage disequilibrium; SNPs, single‐nucleotide polymorphisms; GEO, gene expression omnibus; MR, Mendelian randomization; IVW, inverse variance weighted; WMR, weighted median regression; WMod, weighted mode; SMod, simple mode; PPI, protein–protein interaction; GO, gene ontology; KEGG, Kyoto encyclopedia of genes and genomes.

Using a comprehensive two‐sample MR approach, this study investigated metformin repurposing for PLC treatment to evaluate its pharmacological potential. (Figure 1A) Our MR analysis adhered to three core assumptions: (1) Relevance: Genetic variants must significantly associate with the exposure; (2) Independence: Variants influence outcomes exclusively via exposure, without horizontal pleiotropy; (3) Exclusion restriction: No unmeasured confounders exist between genetic instruments and outcomes (Labrecque and Swanson 2018). First, we evaluated metformin's causal effects on PLC susceptibility—including HCC and ICC. Subsequently, we examined causal effects between genetic variants in metformin‐targeted genes and liver cancer risk among Europeans, to elucidate metformin's potential mechanisms in PLC pathogenesis. Since metformin is primarily indicated for T2D, we first assessed causal effects of genetic variants in metformin‐targeted genes on T2D as an MR‐positive control. Subsequently, bioinformatics analyses elucidated how metformin correlates with liver cancer progression and potential oncogenic mechanisms (Figure 1B).

### 2.2 Data Source for Genome‐Wide Association Study (GWAS)

GWAS identifies genetic variants associated with complex diseases by analyzing population‐scale DNA samples, enabling systematic exploration of genes implicated in disease mechanisms and therapeutic responses. Exposure and outcome datasets were sourced from IEU OpenGWAS (https://gwas.mrcieu.ac.uk/) database. Specifically, the IEU OpenGWAS provided metformin exposure data (11,552 cases and 451,381 controls) and liver cancer outcomes including: Hepatic cancer (379 cases and 475,259 controls), Liver cell carcinoma (168 cases and 372,016 controls), Liver & bile duct cancer (350 cases and 372,016 controls).

Using the DrugBank (https://go.drugbank.com/) database, we identified metformin's target proteins (Xu et al. 2024). In drug‐target MR we restricted metformin ‘targets’ to DrugBank‐curated proteins with strong liver‐relevant cis instruments and clear pharmacodynamic specificity (PRKAB, ETFDH, GPD1L), focusing on nodes along the AMPK–redox axis central to its hepatic action. Upstream regulators (e.g., STK11/LKB1, SIRT1), multi‐subunit complexes (mitochondrial complex I), and pharmacokinetic transporters (e.g., OCT1, MATE) were excluded due to weak or nonspecific instruments and high pleiotropy, ensuring MR validity and interpretability. Genetic variants from these loci were sourced from IEU OpenGWAS and served as IVs to simulate pharmacological regulation of the drug targets. The sample sizes for the exposed datasets are as follows: cis‐eQTLs for PRKAB (ENSG00000111725) (Sample Size 31,684), cis‐eQTLs for ETFDH (ENSG00000171503) (Sample Size 26,395), cis‐eQTLs for GPD1L (ENSG00000152642) (Sample Size 31,684). The GWAS data of T2D (42,593 cases and 337,038 controls) were obtained from the FinnGen R10 (https://www.finngen.fi/en) (DF10‐December 18, 2023) database. All analysis data are categorical (qualitative) variables. The FinnGen study is a study that is collecting genetic samples of Finnish residents across the country, combining genomic information with digital health care and registration data (Kurki et al. 2023). This study utilized exclusively public GWAS data; therefore, neither ethical approval nor participant informed consent was required. Detailed dataset characteristics are presented in Table 1.

**TABLE 1:** Studies used to retrieve summary statistics for the two‐sample Mendelian randomization analyses.

Trait	Outcome/Exposure	Population	Sample size	SNPs	Data source	Dataset	Year
PRKAB	Exposure	European	31,684	18,000	IEU OpenGWAS	eqtl‐a‐ENSG00000111725	2018
ETFDH	Exposure	European	26,395	17,243	IEU OpenGWAS	eqtl‐a‐ENSG00000171503	2018
GPD1L	Exposure	European	31,684	20,081	IEU OpenGWAS	eqtl‐a‐ENSG00000152642	2018
Metformin	Exposure	European	11,552 cases and 451,381 controls	9,851,867	IEU OpenGWAS	ukb‐b‐14,609	2018
T2D	Outcome	European	42,593 cases and 337,038 controls	21,305,664	FinnGen R10	T2D_WIDE	2023
Hepatic cancer	Outcome	European	379 cases and 475,259 controls	24,194,938	IEU OpenGWAS	ebi‐a‐GCST90018858	2021
Liver cell carcinoma	Outcome	European	168 cases and 372,016 controls	6,304,034	IEU OpenGWAS	ieu‐b‐4953	2021
Liver & bile duct cancer	Outcome	European	350 cases and 372,016 controls	7,687,713	IEU OpenGWAS	ieu‐b‐4915	2021

Abbreviations: SNP, single nucleotide polymorphisms; T2D, Type 2 diabetes.

### 2.3 Data Screening

IVs were selected through sequential criteria: first, single nucleotide polymorphisms (SNPs) meeting genome‐wide significance (*p* < 5 × 10^−8^) for exposure associations were identified; second, linkage disequilibrium (LD) clumping using the 1000 Genomes European reference (R^2^ < 0.001, distance = 10,000 kb) ensured independence of variants across distinct loci; finally, summary statistics for exposure‐associated IVs were extracted. To ensure result accuracy, we excluded SNPs in palindromic sequences and those associated with potential confounders. Additionally, the strength of genetic instruments was quantified using the F‐statistic (F = β^2^/SE^2^), with F > 10 indicating reliable instruments. These criteria were applied to identify more reliable IVs, enhancing the overall reliability of the study. Notably, for ETFDH (eQTL‐a‐ENSG00000171503)—a metformin target protein—no IVs met the initial significance threshold (*p* < 5 × 10^−8^; LD *r* ^2^ < 0.001, distance = 10,000 kb). Consequently, we relaxed the criteria to *p* < 5 × 10^−8^, *r* ^2^ < 0.01, and clumping distance = 5000 kb for this exposure.

### 2.4 MR Analysis and Statistical Analysis

Statistical analyses were conducted in R (v4.3.1), employing the ‘TwoSampleMR’ (version 0.6.6) and ‘MRPRESSO’ (version 1.0) packages for Mendelian randomization analyses, and ‘forestploter’ (version 1.1.2) for visualization. Primary causal inference employed inverse‐variance weighted (IVW) regression with fixed effects, aggregating instrumental variables through inverse‐variance weighting. Supplementary analyses included weighted median regression (WMR), MR‐Egger, weighted mode (WMod), and simple mode (SMod) to address distinct validity aspects: IVW assumes all IVs are valid for weighted average estimation; MR‐Egger corrects horizontal pleiotropy via intercept adjustment; WMR maintains robustness with ≤ 50% invalid instruments using median weighting; WMod prioritizes modal estimates from valid IVs; SMod identifies unweighted frequentist ratios. Statistical significance was defined as *p* < 0.05, with effects reported as odds ratios (OR) and 95% confidence intervals (CI). To correct for multiple testing, Benjamini‐Hochberg false discovery rate (FDR) correction was applied. The *p* value threshold < 0.05 after FDR correction (referred to as the P_fdr_) was used to define “strong evidence” supporting the analysis, while findings with P_fdr_ ≥ 0.05 and < 0.20 were defined as “suggestive evidence.”

### 2.5 Sensitivity Analysis

Heterogeneity was assessed via Cochran's Q test (*p* < 0.05 indicating significance). When detected, random‐effects IVW models were employed; otherwise, fixed‐effects models were applied. Horizontal pleiotropy was evaluated using MR‐Egger intercept tests. Potential outliers were identified through MR‐PRESSO, with significant results triggering re‐analysis. Leave‐one‐out sensitivity analyses examined individual SNP influence. Visualization included scatter and funnel plots for outcome interpretation and outlier detection.

### 2.6 Metformin‐PLC Data Sources and Differential Expression Analysis

The Gene Expression Omnibus (GEO; http://www.ncbi.nlm.nih.gov/geo) provided gene expression profiles for this study. Dataset GSE241466 (Platform GPL24676), containing PLC and normal liver tissues, was analyzed using GEO2R. Differentially expressed genes (DEGs) were identified with thresholds of |log2FC| > 0 and *p* < 0.05. We additionally annotated proximal genes within 500 kb of 46 metformin‐associated SNPs. Venn analysis integrated PLC and metformin DEGs for subsequent investigation.

### 2.7 Functional Enrichment Analysis: Protein–Protein Interactions (PPI), Gene Ontology (GO), and Kyoto Encyclopedia of Genes and Genomes (KEGG) Pathways

The PPI network for DEGs was constructed using GeneMANIA (http://www.genemania.org), an online platform for network generation, gene function prediction, and functional similarity identification. Protein‐binding interactions were further analyzed via the STRING database (https://string‐db.org/). Among 34 shared genes, we focused on 10 highly interconnected hub genes. GO enrichment analysis of these hub genes was performed in R Studio using ‘clusterProfiler’ (version 3.19.1), ‘http://org.Hs.eg.db’ (version 3.19.1), ‘ggplot2’ (version 3.5.1) and ‘enrichplot’ (version 1.24.4) packages. KEGG pathway analysis utilized Sangerbox (http://sangerbox.com/), with statistical significance defined as *p* < 0.05.

### 2.8 Single‐Cell Immune Profiling and Tumor Microenvironment (TME) Infiltration Analysis

Due to the scarcity of comprehensive data on PLC and the predominance of HCC as its major subtype, HCC‐specific data were used for subsequent analyses.

GEPIA2 (http://gepia.cancer‐pku.cn/), an interactive web server integrating the Cancer Genome Atlas (TCGA) and Genotype‐Tissue Expression (GTEx) data (9736 tumors; 8587 normals), was employed to analyze survival‐associated DEGs. From the 34 candidate genes, we identified TICRR, DDX52, KIF11, GCDH, and MRPL45 as significantly correlated with HCC overall survival (OS) and disease‐free survival (DFS). Validation of DEGs using liver hepatocellular carcinoma (LIHC) data from TCGA (https://www.cancer.gov/ccg/). The Tumor Immune Single‐cell Hub (TISCH; http://tisch.comp‐genomics.org/) integrates single‐cell RNA sequencing data for systematic TME heterogeneity exploration. Using its LIHC_GSE140228_10X dataset, we investigated associations between differentially expressed genes and TME components.

We further employed the Tumor IMmune Estimation Resource database (TIMER; https://cistrome.shinyapps.io/timer/) to assess associations between candidate genes and infiltration levels of six immune cell types: CD4^+^/CD8^+^ T cells, neutrophils, myeloid dendritic cells, macrophages, and B cells. Due to unavailable TICRR data in TIMER, analyses focused on DDX52, KIF11, GCDH, and MRPL45 correlations with these immune cell subtypes.

### 2.9 Immunotherapy and Drug Sensitivity Analysis

To assess the immunotherapy response of four DEGs in liver cancer, we investigated their associations with immune checkpoint inhibitors (ICIs) using TIMER. Expression correlations between these DEGs and key checkpoints—programmed death‐1 (PD‐1 or PDCD1), programmed death‐ligand 1 (PD‐L1 or CD274), and CTLA4—were visualized via scatter plots. For drug sensitivity prediction, the Gene Set Cancer Analysis platform (GSCA; https://guolab.wchscu.cn/GSCA) integrated Genomics of Drug Sensitivity in Cancer (GDSC) data to evaluate DDX52, KIF11, GCDH, and MRPL45 (TICRR data unavailable). Sensitive drug structures were identified through DrugBank.

## 3 Results

### 3.1 Causal Effects of Metformin on PLC, HCC, and ICC

**FIGURE 2:**
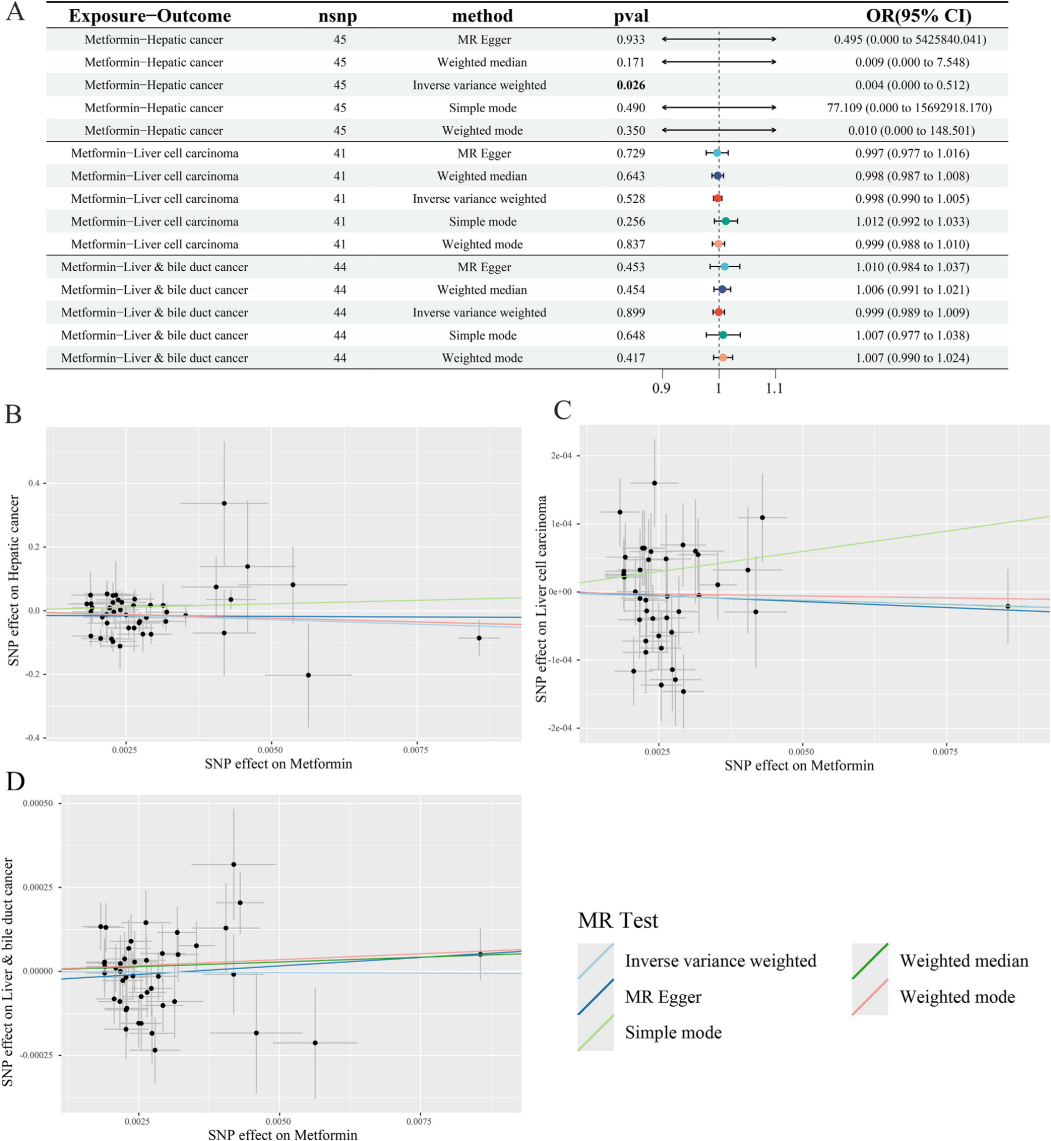
Associations of genetically predicted metformin with risk of liver cancer using the MR method. (A) A forest plot illustrates the connections between metformin and the risk of liver cancer. (B–D) Scatter plots for the causal association between metformin and liver cancer. MR, Mendelian randomization.

A total of 46 index SNPs were identified as metformin‐associated instrumental variables (IVs). All IVs demonstrated adequate strength, with F‐statistics ranging from 29.75 to 997.38. The detailed characteristics of metformin‐related IVs are presented in Table S1, and the integration of SNP data across metformin and liver cancer datasets is summarized in Table S2. A rigorous two‐sample MR analysis investigated metformin's causal effects on PLC. IVW estimates demonstrated metformin's protective association with hepatic cancer risk (*β* = −5.6046, OR = 0.0037, *p* = 0.026) (Figure 2A). FDR correction of MR estimates shows P_fdr_ > 0.05 (Table S3). Although the MR analysis for metformin and PLC yielded a *p* value below the conventional threshold of 0.05, the associated FDR exceeded this cutoff. In this study, we prioritized *p* value interpretation in light of the specific analytical context. While recognizing the value of FDR correction for multiple comparisons, we considered the uncorrected *p* value to remain informative for a conservative assessment of statistical significance. Moreover, the weighted median, simple mode, and weighted mode methods all exhibited effect estimates directionally consistent with the IVW approach, despite lacking uniform statistical significance. Further analyses of HCC and ICC subtypes revealed non‐significant causal relationship via IVW (HCC: *β* = −0.0024, OR = 0.9976, *p* = 0.528; ICC: *β* = −0.0007, OR = 0.9993, *p* = 0.899) (Table S3, Figure 2A). Although MR‐Egger, WMR, and WMod methods yielded *p* > 0.05, their β‐direction consistently aligned with IVW (Figure 2B–D). Scatter plots and leave‐one‐out plots visualize these relationships (Figure 2B–D, Figure S1). The differential effect of metformin on reducing the overall risk of PLC and HCC/ICC may stem from its greater propensity to intervene in the progression of precancerous liver disease, or from insufficient statistical power in the HCC subtype analysis within this study.

Sensitivity analysis indicates, MR‐Egger intercept tests yielded non‐significant results (all *p* > 0.05), indicating no horizontal pleiotropy (Table 2). Cochran's Q test revealed significant heterogeneity for metformin associations with PLC, HCC, and ICC (*p* < 0.05). This heterogeneity may reflect population‐specific genetic effects or environmental interactions. Crucially, our application of random‐effects IVW models mitigated heterogeneity‐induced bias (Papadimitriou et al. 2020). Additionally, MR‐PRESSO and leave‐one‐out analyses identified no significant outliers.

**TABLE 2:** Sensitivity analysis, heterogeneity, and pleiotropy, investigating MR assumption violatio.

Exposures‐Outcome	Heterogeneity tests				MR‐PRESSO		Egger intercept	
	IVW	*p* value	MR Egger	*p* value	Outliers	*p* value	Intercept	*p* value
metformin‐Hepatic cancer	63.074661	0.031	62.511	0.027	0	0.021	‐1.42E‐02	0.537
metformin‐Liver cell carcinoma	61.840729	0.015	61.818	0.011	0	0.017	3.41E‐06	0.906
metformin‐Liver & bile duct cancer	61.629297	0.033	60.527	0.032	0	0.038	‐3.38E‐05	0.387
PRKAB‐Type 2 diabetes	6.576	0.254	5.230	0.265	0	0.380	2.58E‐02	0.368
ETFDH‐Type 2 diabetes	0.948	0.918	0.944	0.815	0	0.878	4.95E‐04	0.955
GPD1L‐Type 2 diabetes	6.599	0.086	2.608	0.271	0	0.386	‐1.67E‐02	0.222
PRKAB‐Hepatic cancer	5.472	0.485	5.469	0.361	0	0.466	1.03E‐02	0.960
ETFDH‐Hepatic cancer	3.351	0.501	0.721	0.868	0	0.685	5.83E‐02	0.203
GPD1L‐Hepatic cancer	3.915	0.271	2.905	0.234	0	0.486	3.06E‐02	0.492

Abbreviations: IVW, Inverse Variance Weighted; MR, Mendelian randomization.

### 3.2 Causal Effects of Metformin Drug Targets on T2D and Hepatic Cancer

Three metformin targets—PRKAB, ETFDH, and GPD1L—were retrieved from DrugBank. We identified 7, 5, and 4 IVs associated with PRKAB, ETFDH, and GPD1L, respectively. All IVs demonstrated robustness (F‐statistics = 34.424–2183.604; all > 10). Detailed SNP characteristics are documented in Tables S4 and S5.

**FIGURE 3:**
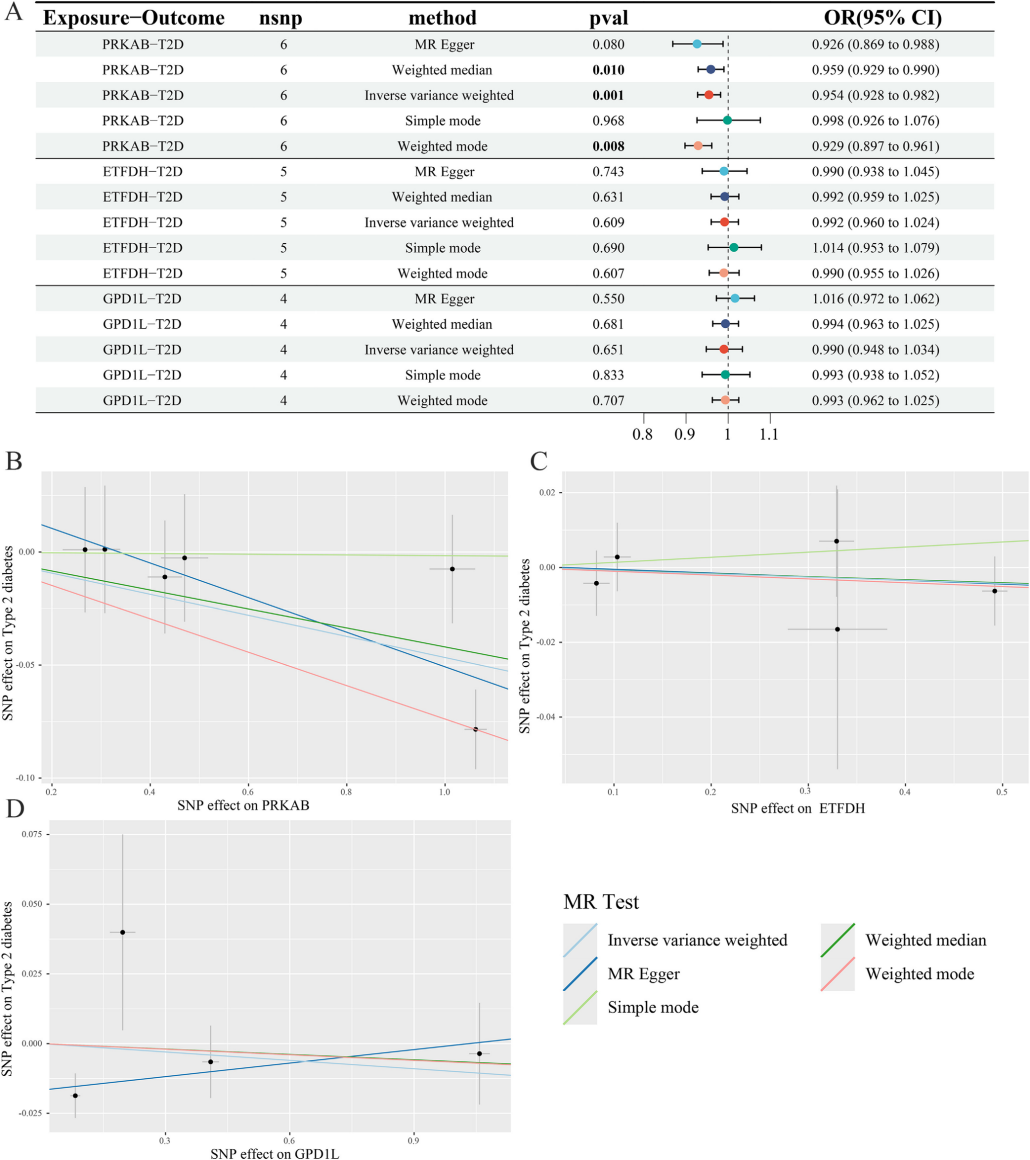
Associations of genetically predicted drug targets of metformin with the risk of T2D using the MR method. (A) A forest plot illustrates the connections between the targets and the risk of T2D. (B–D) Scatter plots for the causal association between the targets and T2D. MR, Mendelian randomization; T2D, type 2 diabetes.

Table S6 presents MR analyses of metformin targets and T2D. IVW estimates showed that PRKAB upregulation—associated with AMPK activity—significantly reduced T2D risk [OR = 0.954, 95% CI 0.928–0.982; *p* = 0.001], FDR correction of MR estimates shows P_fdr_ < 0.05 (Table S7). No significant causal effects were observed for functional inhibition of ETFDH [OR = 0.992, 95% CI 0.960–1.024; *p* = 0.609] or GPD1L [OR = 0.990, 95% CI 0.948–1.034; *p* = 0.651] (Figure 3A, Table S7). Effect estimates across MR methods are visualized in scatter and leave‐one‐out plots (Figure 3B–D, Figure S2).

**FIGURE 4:**
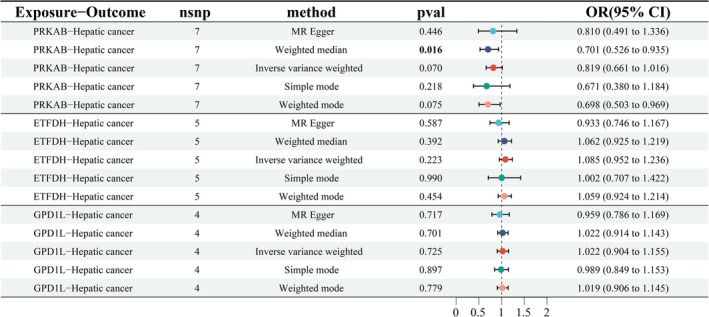
Associations of genetically predicted drug targets of metformin with the risk of hepatic cancer using the MR method. A forest plot illustrates the connections between the targets and the risk of hepatic cancer. MR, Mendelian randomization.

Building on established metformin‐ hepatic cancer causality, MR analysis of drug targets (Table S8) revealed non‐significant causal effects via IVW: PRKAB (OR = 0.819, 95% CI 0.661–1.016, *p* = 0.070), ETFDH (OR = 1.085, 95% CI 0.952–1.236, *p* = 0.223), and GPD1L (OR = 1.022, 95% CI 0.904–1.155, *p* = 0.725), potentially due to limited statistical power (Figure 4). Notably, all methods (MR‐Egger/WMR/IVW/SMod/WMod) consistently demonstrated negative *β*‐values for PRKAB‐HCC causal effects, suggesting its potential role in metformin's adjuvant therapeutic mechanisms.

Sensitivity analyses showed that the MR‐Egger intercept test was non‐significant (*p* > 0.05), suggesting the absence of horizontal pleiotropy. Moreover, MR‐PRESSO, leave‐one‐out analyses, and Cochran's Q test detected no significant outliers (all *p* > 0.05) (Table 2).

### 3.3 DEGs Analysis in HCC and Metformin SNPs

**FIGURE 5:**
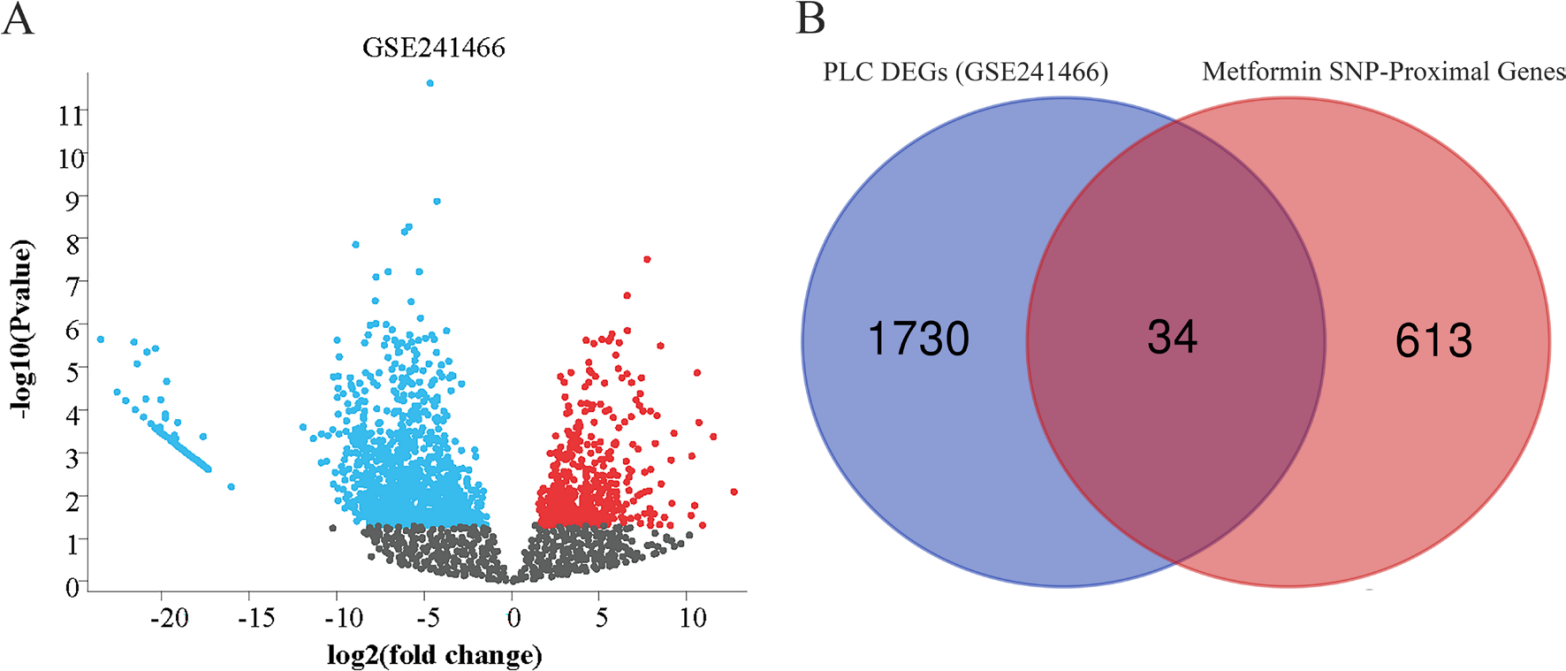
Differential expression analysis of genes in PLC and metformin datasets: (A) Volcano plot of differentially expressed genes in GSE241466 and (B) Venn diagram of differentially expressed genes in metformin datasets and PLC (GSE241466). PLC, primary liver cancer.

To identify shared DEGs between PLC and metformin‐associated genomic loci, we analyzed the GSE241466 dataset from GEO. Figure 5A displays the volcano plot visualizing gene dysregulation patterns. Venn diagrams then identified overlapping DEGs between metformin‐related targets and 34 PLC‐associated genes in tumor/paracancerous tissues (Figure 5B).

### 3.4 PPI Analysis and Enrichment Pathway Analysis

**FIGURE 6:**
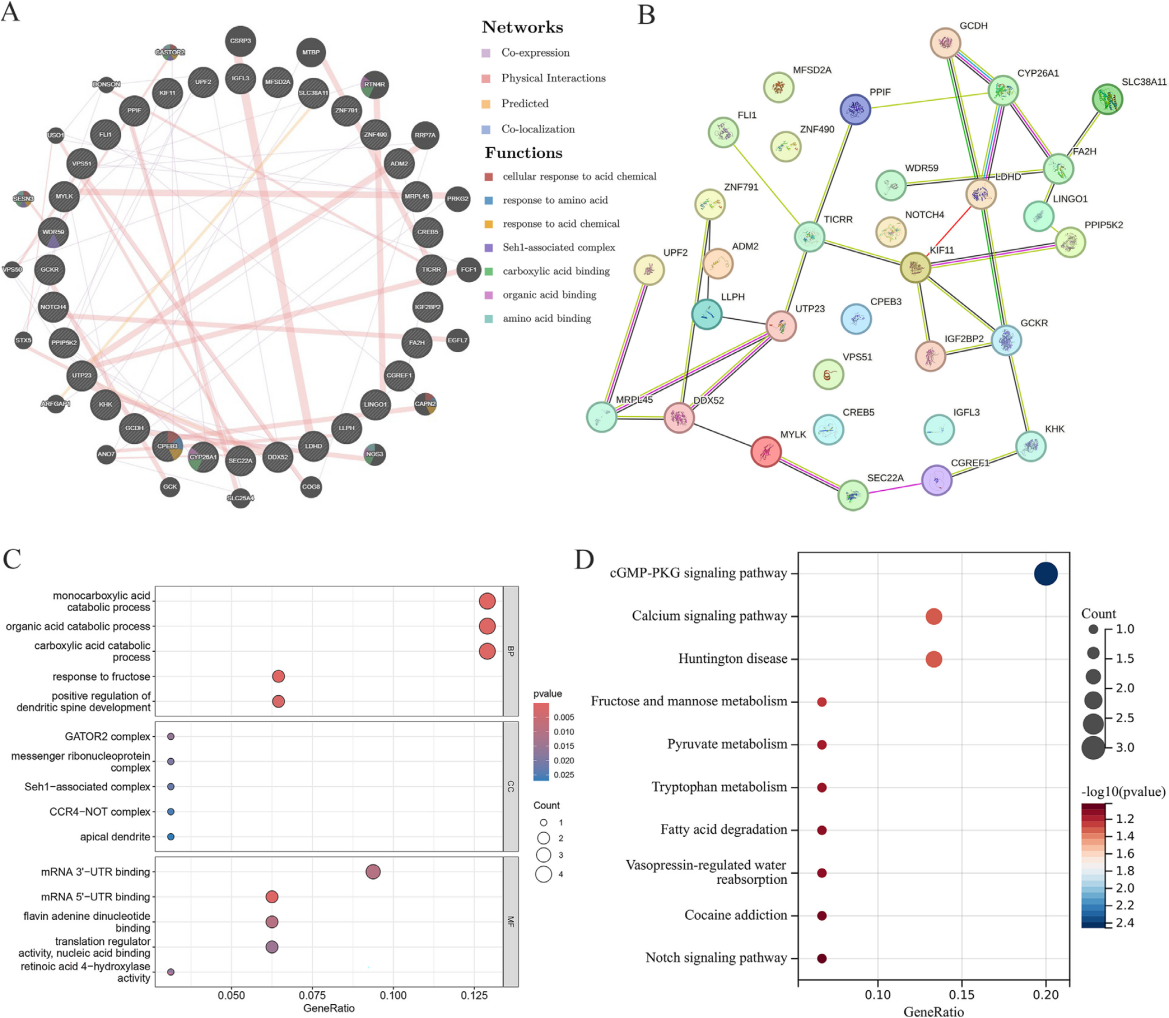
PPI and functional enrichment pathway analysis. (A) A PPI network of differentially expressed genes using GeneMANIA. (B) A PPI network of differentially expressed genes and closely related genes using STRING. (C) GO enrichment analysis results of differentially expressed genes and closely related genes. (D) KEGG pathway analysis results of differentially expressed genes and closely related genes. PPI, protein–protein interaction; GO, gene ontology; KEGG, Kyoto encyclopedia of genes and genomes.

To elucidate the biological significance of these DEGs in PLC, we identified interacting proteins and performed pathway enrichment analysis, constructing a PPI network through GeneMANIA and STRING (Figure 6A,B). Simultaneously, we discovered that these genes are associated with processes such as cellular response to acid chemical, response to amino acid, response to acid chemical, Seh1‐associated complex, carboxylic acid binding, organic acid binding, amino acid binding. Subsequently, we conducted GO and KEGG analyses on the differentially expressed genes to further explore the molecular mechanisms through which metformin exerts its protective effects on PLC. As shown in Figure 6C, GO enrichment analysis revealed predominant localization of these co‐expressed genes in Cellular Components (CCs), specifically the GATOR2 and messenger ribonucleoprotein complexes. Within Biological Process (BP) ontologies, these co‐expressed genes demonstrated primary enrichment in monocarboxylic and organic acid catabolism. Within Molecular Function (MF) ontologies, these co‐expressed genes primarily functioned in mRNA 3ʹ‐UTR and 5ʹ‐UTR binding. KEGG pathway analysis revealed that these differentially expressed genes were closely related to the cGMP‐PKG signaling pathway, calcium signaling pathway, Huntington's disease, and fructose and mannose metabolism (Figure 6D).

### 3.5 Correlation Between DEGs and the TME

**FIGURE 7:**
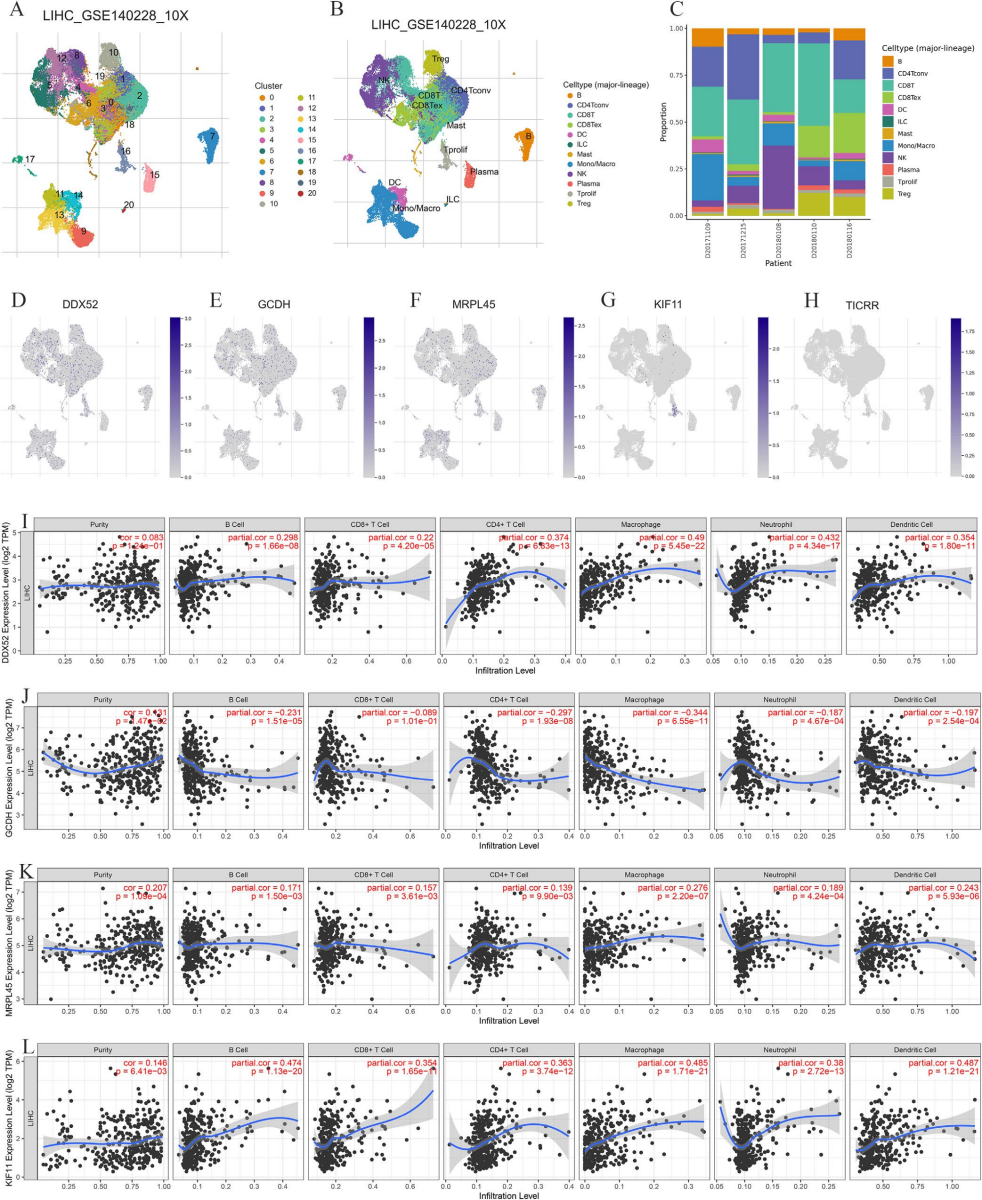
Correlation between differentially expressed genes and TME at the single‐cell level. (A–C) Classification and statistics of cell types in the LIHC_GSE140228_10X data set. (D–H) Distribution and expression of the five differentially expressed genes (DDX52, GCDH, MRPL45, KIF11, and TICRR). (I–L) The correlation between four differentially expressed genes and immune cell infiltration in HCC. TME, tumor microenvironment; HCC, hepatocellular carcinoma.

Using the GEPIA2 database, we identified five prognosis‐associated genes (TICRR, DDX52, KIF11, GCDH, MRPL45) from 34 candidate DEGs that correlate with the OS and DFS of HCC (Figure S3), the TCGA‐LIHC database further confirms the differential expression of these five genes in HCC (Figure S4). Given their potential significance in hepatocarcinogenesis, we conducted focused single‐cell RNA sequencing analysis of these genes via the TISCH database (LIHC_GSE140228_10X dataset). Single‐cell analysis identified 20 distinct clusters, consolidated into 12 major cell types (Figure 7A–C). DDX52 demonstrated ubiquitous high expression across most immune lineages (Figure 7D), while GCDH and MRPL45 showed moderate expression in select immune populations. Conversely, KIF11 and TICRR exhibited negligible expression in these cells (Figure 7E–H). TIMER database analysis (TICRR data unavailable) revealed distinct immune infiltration patterns for four genes in PLC (Figure 7I–L): DDX52, KIF11, and MRPL45 showed significant positive correlations with B cells, CD8^+^/CD4^+^ T cells, macrophages, neutrophils, and dendritic cells, while GCDH exhibited consistent negative correlations with all six immune populations.

### 3.6 Immunotherapeutic Relevance and Pharmacogenomic Sensitivity Profiling

**FIGURE 8:**
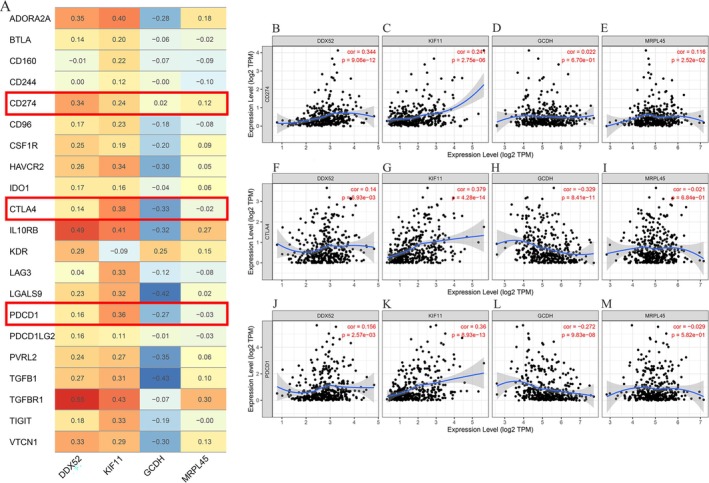
Correlation between differentially expressed genes and immune checkpoint inhibitors in HCC. (A) A heatmap displays the correlation between four differentially expressed genes and immune checkpoint inhibitors in HCC. (B–D) A scatter plot illustrates the correlation between the expression of four differentially expressed genes and PD‐1, PD‐L1, and CTLA‐4. HCC, Hepatocellular carcinoma.

**FIGURE 9:**
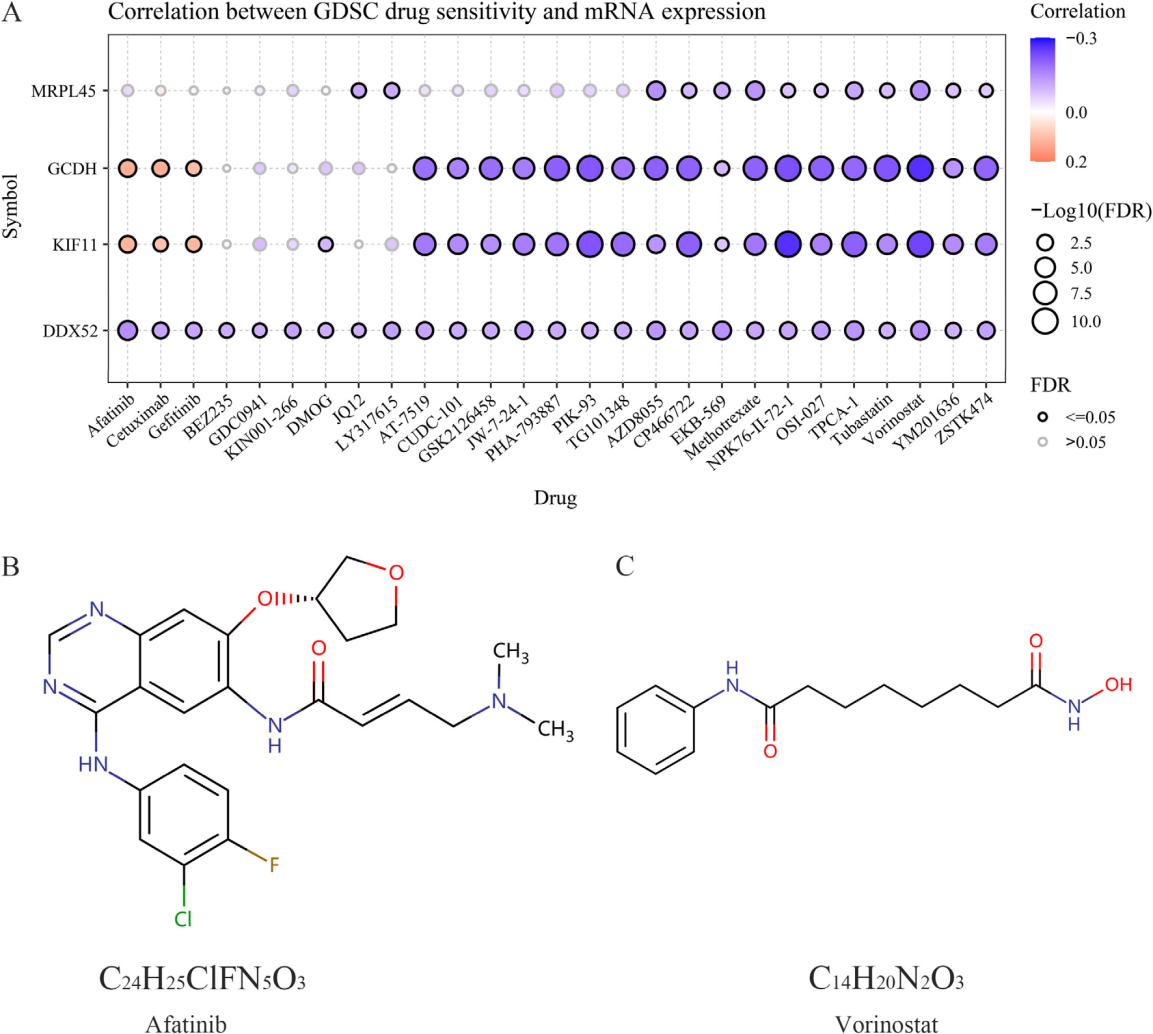
Gene‐drug interaction and drug sensitivity analysis. (A) The connection between the expression of four differentially expressed genes and drug responsiveness in the GSCA database. (B, C) The molecular formulas of Afatinib and Vorinostat.

ICIs are fundamental to tumor immunotherapy. Analysis via the TIMER database indicates that three DEGs—excluding GCDH—exhibit positive correlations with most ICIs expression (Figure 8A). PD‐1 (PDCD1), PD‐L1 (CD274), and CTLA‐4 represent clinically pivotal immune checkpoints. Our findings indicate that CD274 expression correlates positively with DDX52, KIF11, GCDH, and MRPL45, while CTLA4 shows a positive correlation with GCDH and MRPL45. Additionally, PDCD1 expression is positively correlated with GCDH and MRPL45 (Figure 8B–M). Using GDSC data from GSCA (TICRR unavailable), we assessed pharmacogenomic correlations between four DEGs and drug sensitivity (Figure 9A). Results revealed a significant positive correlation between KIF11 expression and Afatinib sensitivity, whereas GCDH showed an inverse correlation with Vorinostat sensitivity. Molecular structures of these agents are provided for reference (Figure 9B,C).

## 4 Discussion

As an established first‐line therapy for T2D mellitus, metformin primarily functions as an antihyperglycemic agent while exerting multifaceted pleiotropic effects across biological systems. Epidemiological evidence since 2005 has ignited interest in its potential anticancer properties, demonstrating inhibitory effects on neoplastic inception and advancement through diverse mechanisms (Choksi et al. 2023). Subsequent studies validate metformin's hepatoprotective efficacy, with accumulating epidemiological evidence associating its use with reduced incidence and mortality across multiple malignancies, particularly PLC (Yang et al. 2021). Despite increasing interest in metformin's potential role in primary liver carcinogenesis, a comprehensive evaluation of this association remains lacking. Most studies to date have been observational, limiting their ability to infer causality due to the presence of residual confounders and the possibility of reverse causality (Mukherjee et al. 2023; Najafi et al. 2023).

To address these limitations, we implemented a two‐sample MR framework to assess the causal effect of metformin exposure on PLC pathogenesis. In this study, we conducted two‐sample MR analyses to assess potential causal relationships between metformin and the risk of PLC and its subtypes in the European population. Our study found a significant correlation between metformin and PLC (*β* = −5.6046, OR = 0.0037, *p* = 0.026), indicating that metformin may play a crucial role in the development of PLC. Although we employed multiple methods to mitigate statistical bias in our study, it must be acknowledged that the effect size OR = 0.0037 may still exhibit weak instrumental variable bias. This necessitates cautious interpretation of its clinical significance. Additionally, two‐sample MR analysis revealed non‐significant causal effects of metformin on HCC or ICC. While metformin reduced overall PLC risk, it did not lower the risk of HCC or ICC, suggesting that metformin's antitumor effects may primarily manifest in inhibiting the malignant transformation of precancerous lesions (such as cirrhosis) rather than directly preventing HCC development. Of course, the limited statistical power of the HCC subgroup analysis is also a potential contributing factor. We subsequently examined causal links between metformin's pharmacological targets and PLC risk, constituting the first MR investigation of genetic proxies for these targets in PLC pathogenesis. From the DrugBank database, we identified three key targets of metformin: PRKAB, ETFDH, and GPD1L. Unfortunately, our study did not establish any significant causal relationships between these targets and PLC (IVW: *p* = 0.070 for PRKAB; IVW: *p* = 0.223 for ETFDH; IVW: *p* = 0.725 for GPD1L). Notably, the causal effect estimates for PRKAB across the MR‐Egger, WMR, IVW, SMod, and WMod methods aligned directionally with those for metformin and PLC, suggesting a potential link. PRKAB, also known as the β‐1 subunit of 5ʹ AMP‐activated protein kinase (AMPK), is critical for metformin's effects. Beyond its established function in glycometabolic regulation, AMPK serves as a pivotal mediator of metformin's oncostatic effects (Triggle et al. 2022). Therefore, we hypothesize that PRKAB may also serve as a key target for metformin's antagonism against PLC; however, research in this area remains limited and requires further investigation to confirm.

Oncogenesis can arise from the inactivation of tumor‐suppressor genes and the overexpression of oncogenes (Chen et al. 2022). In this study, we identified five DEGs in PLC—GCDH, MRPL45, DDX52, KIF11, and TICRR—mapped from metformin‐associated SNPs. These genes may be crucial for elucidating the potential protective mechanisms of metformin in PLC. PPI network enrichment analysis indicated that they are involved in cellular responses to acidic chemicals and to amino acids, are associated with the Seh1‐associated complex, and participate in the regulation of carboxylic acid, organic acid, and amino acid binding. Zhou et al. (2023) reported that the anticancer activity of metformin in malignant cells is primarily mediated by activation of AMPK, which in turn engages multiple downstream pathways that cooperatively suppress tumor growth, The AMPK‐driven mechanisms include inhibition of mammalian target of rapamycin (mTOR) and modulation of key regulators such as Cyclin D1, p53, p21, p27, and Akt, thereby exerting antiproliferative effects. In line with this, Choksi et al. (2023) showed that combining metformin with locoregional therapy produced notable benefits, including improved overall survival, reduced tumor recurrence, decreased cellular proliferation and migration, and increased DNA damage and apoptosis. Based on these observations, we hypothesize that the metformin‐related differentially expressed genes may influence HCC progression through cellular regulatory processes. KEGG analysis indicated enrichment of the cGMP‐PKG signaling pathway, which has been implicated in antiproliferative and proapoptotic effects as well as the regulation of angiogenesis. Within this framework, we propose that the metabolism‐associated genes GCDH (involved in fatty acid oxidation) and MRPL45 (essential for the synthesis of oxidative phosphorylation complexes) function as key sensors of mitochondrial metabolic state, with their altered expression reflecting metformin‐induced metabolic reprogramming. We further posit that this remodeled metabolic state signals through the cGMP‐PKG axis. On one hand, it may directly inhibit phospholipase C activity, reducing lactate accumulation and the stability of HIF 1α, improving oxygenation in the tumor microenvironment, diminishing invasive and metastatic potential, and thereby restraining tumor growth (Lao et al. 2024). On the other hand, PKG activation can modulate T cell receptor signaling, cytokine production, and chemotactic responses, fostering an immune milieu that supports the survival, fitness, and effector function of CD8^+^ T cells. Consistently, Wabitsch et al. demonstrated that metformin induces metabolic reprogramming of hepatic CD8^+^ T cells, increasing the motility of these tumor‐infiltrating lymphocytes and enhancing antitumor efficacy, concordant with our observation of a positive association between metformin exposure and CD8^+^ T cell infiltration (Wabitsch et al. 2022). In summary, metformin may suppress tumor growth by inhibiting PLC via GCDH/MRPL45‐mediated fatty acid oxidation and activating the cGMP‐PKG pathway, although this putative mechanism remains to be validated by further studies.

The TME dynamically coordinates fundamental cancer hallmarks: hyperproliferation, apoptosis evasion, angiogenic switching, metastatic progression, inflammation‐driven tumorigenesis, and immune escape. Additionally, the composition of immune cells within the TME is essential for tumor progression (Du et al. 2021). Our study reveals that genes differentially co‐expressed with metformin proximal SNPs and PLC show strong correlations with immune cell infiltration. Beyond GCDH, MRPL45, KIF11, and DDX52 all exhibit positive correlations with CD4^+^/CD8^+^ T cells, neutrophils, myeloid dendritic cells, macrophages, and B cells. This suggests that cis‐regulatory effects of metformin‐associated SNPs may modulate immune cell infiltration in PLC. Upregulation of these differentially expressed genes promotes immune cell recruitment to the TME, fostering an “immunologically active” tumor phenotype. Among these genes, GCDH and MRPL45 are key regulators of mitochondrial energy metabolism and oxidative phosphorylation. We posit that, by driving metabolic reprogramming, they may shift tumor cells toward greater reliance on OXPHOS, thereby reducing lactate production in the TME, alleviating acidosis, and releasing the metabolic constraints on immune cell function, collectively suppressing tumor progression (Ye et al. 2022). KIF11, a critical mitotic motor protein, is closely associated with cellular proliferation (Chinen et al. 2020). Guo et al. (2022) reported that KIF11 upregulation promotes HCC progression and portends poor prognosis, consistent with its role in driving mitosis and proliferation in malignant cells. Complementarily, You et al. showed that metformin inhibits the proliferation of HCC cells and arrests the cell cycle at G0/G1 (You et al. 2020). In our analysis, KIF11 expression correlated positively with immune cell infiltration, raising the possibility that KIF11 may also facilitate immune cell mitosis and thereby contribute to anti‐tumor immunity. These observations suggest a context‐dependent dual role for KIF11 in regulating mitosis in tumor versus immune cells, which merits further investigation. DDX52, an RNA helicase, may participate in ribosomal RNA processing and in the regulation of innate immune signaling pathways (Xu and Yang 2023). Its upregulation could enhance the expression of interferon‐stimulated genes and promote antigen presentation mechanisms, for example through major histocompatibility complex class I, rendering tumor cells more readily recognized and eliminated by immune cells. Additionally, we observed significant positive correlations between the expression levels of these DEGs and common immune checkpoint markers, suggesting that metformin may enhance responsiveness to ICIs. Consistent with this notion, Wei et al. reported that macrophage‐derived microparticles loaded with metformin targeted M2‐like tumor‐associated macrophages and augmented the efficacy of anti‐PD1 antibody therapy (Wei et al. 2021). Nevertheless, how metformin‐related changes in gene expression modulate immune cell infiltration and immunotherapy response remains insufficiently understood, and this hypothesis requires further validation with larger clinical datasets.

## 5 Limitations

This study has several limitations. First, our exposure and instruments were derived from the UK Biobank “metformin” phenotype and cis eQTLs for PRKAB, ETFDH, and GPD1L, which proxy prescribing or target biology rather than dose or pharmacokinetics; real‐world effects may therefore be under captured. Second, the FDR‐corrected *p* value for metformin on PLC is P_fdr_ = 0.130, which should be considered suggestive. Third, small case counts (HCC *n* = 168; hepatic cancer *n* = 379) limited power for primary and target‐based MR. Fourth, our GWAS samples were predominantly European, constraining generalizability. Finally, bioinformatics results rely on a single GEO dataset (GSE241466), correlative single‐cell/TME and checkpoint analyses (GEPIA2, TISCH, TIMER), and cell‐line drug‐sensitivity data (GSCA/GDSC), all of which require external validation. To overcome these limitations, conducting prospective, multicenter, large‐sample clinical and basic research would be highly beneficial. Such study designs can yield more robust and reliable results, thereby providing strong evidence to further confirm metformin's inhibitory effect on liver cancer progression.

## 6 Conclusion

In conclusion, this study reveals a significant association between metformin and a reduced risk of PLC, but no significant association with a reduced risk of HCC or ICC. This suggests its potential as an adjunct therapy for PLC, though clinical interpretation requires caution. Bioinformatics analysis identified key DEGs in HCC, such as DDX52, KIF11, GCDH, and MRPL45, which are associated with the effects of metformin on HCC. These genes are involved in crucial pathways, including the cGMP‐PKG signaling and fatty acid metabolism pathways, indicating their role in regulating tumor progression. Further research should explore these molecular mechanisms to fully understand how metformin influences PLC development and improve therapeutic strategies.

## Author Contributions

**Yongxin Ma:** conceptualization (equal), data curation (equal), methodology (equal), software (equal), validation (equal), visualization (equal), writing – original draft (equal). **Jiaojiao Qi:** conceptualization (equal), data curation (equal), writing – original draft (equal). **Zhiqiang Chen:** data curation (equal), investigation (equal), visualization (equal). **Yubo Zhang:** methodology (equal), software (equal), visualization (equal). **Kejun Liu:** data curation (equal), investigation (equal). **Jiaxin Suo:** validation (equal), visualization (equal). **Bendong Chen:** methodology (equal), project administration (equal). **Yang Bu:** methodology (equal), project administration (equal).

## Conflicts of Interest

The authors declare no conflicts of interest.

## Supporting information


**Figure S1:** Associations of genetically predicted metformin with risk of liver cancer using the MR method. (A) MR leave‐one‐out sensitivity analysis for metformin on hepatic cancer. (B) MR leave‐one‐out sensitivity analysis for metformin on liver cell carcinoma. (C) MR leave‐one‐out sensitivity analysis for metformin on liver & bile duct cancer. *MR* Mendelian randomization.
**Figure S2:** Associations of genetically predicted the drug targets of metformin with risk of T2D using the MR method. (A) MR leave‐one‐out sensitivity analysis for PRKAB on T2D. (B) MR leave‐one‐out sensitivity analysis for ETFDH on T2D. (C) MR leave‐one‐out sensitivity analysis for GPD1L on T2D. *MR* Mendelian randomization. *T2D* type 2 diabetes.
**Figure S3:** Association between shared genes and survival in metformin dataset and PLC dataset. (A) Association between shared genes and DFS. (B) Relationship between shared genes and OS. *PLC* primary liver cancer *DFS* Disease Free Survival. *OS* Overall Survival.

**Figure S4:** Expression of TICRR, DDX52, KIF11, GCDH, and MRPL45 in the TCGA‐LIHC dataset. (A) The expression of TICRR in the TCGA‐LIHC dataset. (B) The expression of DDX52 in the TCGA‐LIHC dataset. (C) The expression of KIF11 in the TCGA‐LIHC dataset. (D) The expression of GCDH in the TCGA‐LIHC dataset. (E) The expression of MRPL45 in the TCGA‐LIHC dataset.

**Table S1:** SNPs were used as IVs from metformin.
**Table S3:** The MR study on metformin and hepatic cancer and liver cell carcinoma and liver & bile duct cancer.
**Table S4:** SNPs were used as IVs from metformin drug targets.
**Table S5:** Integration of SNPs data from metformin drug targets with hepatic cancer.
**Table S6:** Integration of SNPs data from metformin drug targets with T2D.
**Table S7:** The MR study on metformin drug targets and T2D.
**Table S8:** The MR study on metformin drug targets and hepatic cancer.

## Data Availability

All data used in this study are included in this paper or uploaded as Supporting Information, and public data are available on public platforms. No other unpublished data are available for this study. Software code and data are available from the corresponding author upon request.
