# Supplementary material for: Mendelian Randomization and Bioinformatics Analysis Reveal the Potential Protective Role of Metformin in Primary Liver Cancer
Source: Food Sci Nutr. 2025 Nov 2;13(11):e71156. doi: 10.1002/fsn3.71156 (PMC12580285; doi:10.1002/fsn3.71156)
Supplement: Supplementary file 1 — Figure S1: Associations of genetically predicted metformin with risk of liver cancer using the MR method. (A) MR leave‐one‐out sensitivity analysis for metformin on hepatic cancer. (B) MR leave‐one‐out sensitivity analysis for metformin on liver cell carcinoma. (C) MR leave‐one‐out sensitivity analysis for metformin on liver & bile duct cancer. MR Mendelian randomization. Figure S2: Associations of genetically predicted the drug targets of metformin with risk of T2D using the MR method. (A) MR leave‐one‐out sensitivity analysis for PRKAB on T2D. (B) MR leave‐one‐out sensitivity analysis for ETFDH on T2D. (C) MR leave‐one‐out sensitivity analysis for GPD1L on T2D. MR Mendelian randomization. T2D type 2 diabetes. Figure S3: Association between shared genes and survival in metformin dataset and PLC dataset. (A) Association between shared genes and DFS. (B) Relationship between shared genes and OS. PLC primary liver cancer DFS Disease Free Survival. OS Overall Survival. [file FSN3-13-e71156-s003.docx]

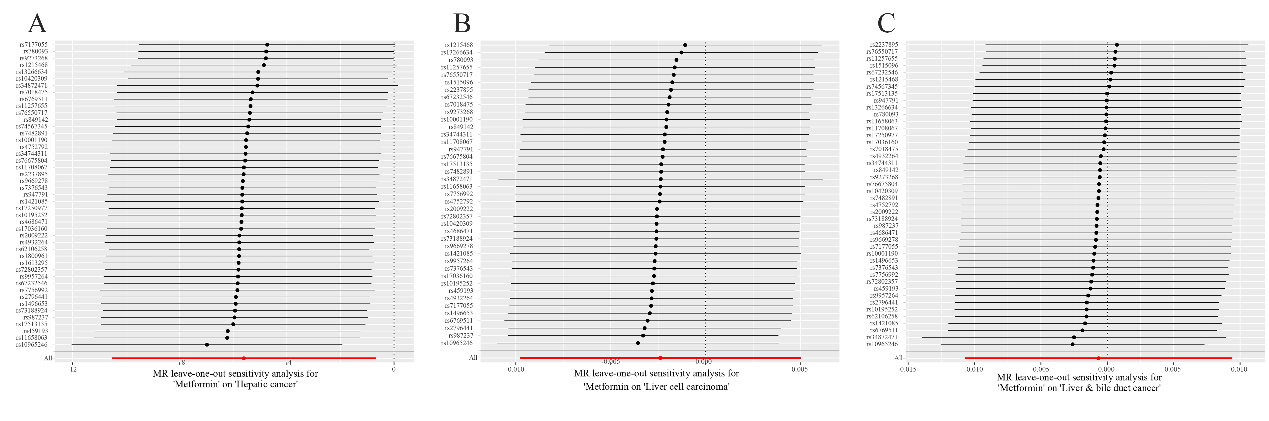


**Figure S1: Associations of genetically predicted metformin with risk of liver cancer using the MR method.** (A) MR leave-one-out sensitivity analysis for metformin on hepatic cancer. (B) MR leave-one-out sensitivity analysis for metformin on liver cell carcinoma. (C) MR leave-one-out sensitivity analysis for metformin on liver & bile duct cancer. *MR* Mendelian randomization.


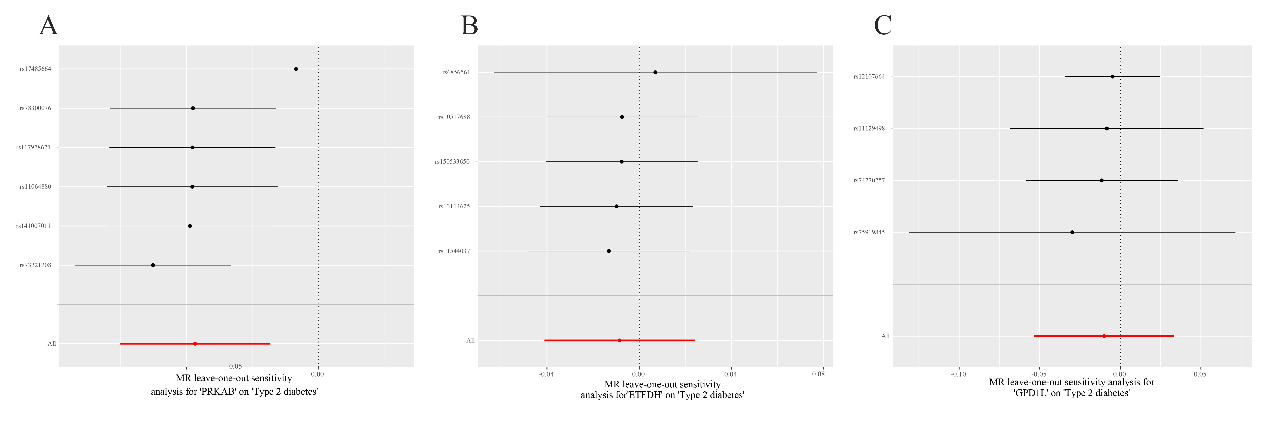


**Figure S2: Associations of genetically predicted the drug targets of metformin with risk of T2D using the MR method.** (A) MR leave-one-out sensitivity analysis for PRKAB on T2D. (B) MR leave-one-out sensitivity analysis for ETFDH on T2D. (C) MR leave-one-out sensitivity analysis for GPD1L on T2D. *MR* Mendelian randomization. *T2D* type 2 diabetes


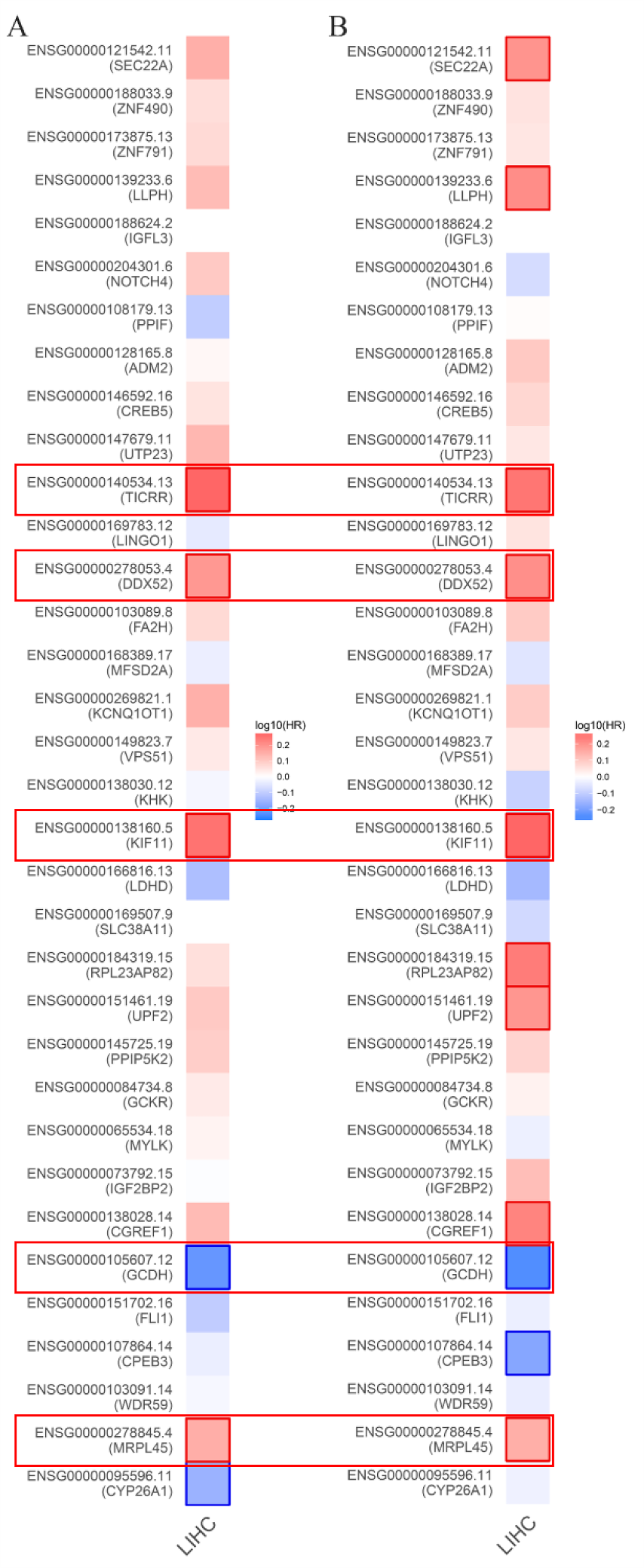


**Figure S3: Association between shared genes and survival in metformin dataset and PLC dataset.** (A) Association between shared genes and DFS. (B) Relationship between shared genes and OS. *PLC* primary liver cancer. *PLC* primary liver cancer *DFS* Disease Free Survival. *OS* Overall Survival.
