# Supplementary figures and images for: Mendelian Randomization and Bioinformatics Analysis Reveal the Potential Protective Role of Metformin in Primary Liver Cancer
Source: Food Sci Nutr. 2025 Nov 2;13(11):e71156. doi: 10.1002/fsn3.71156 (PMC12580285; doi:10.1002/fsn3.71156)

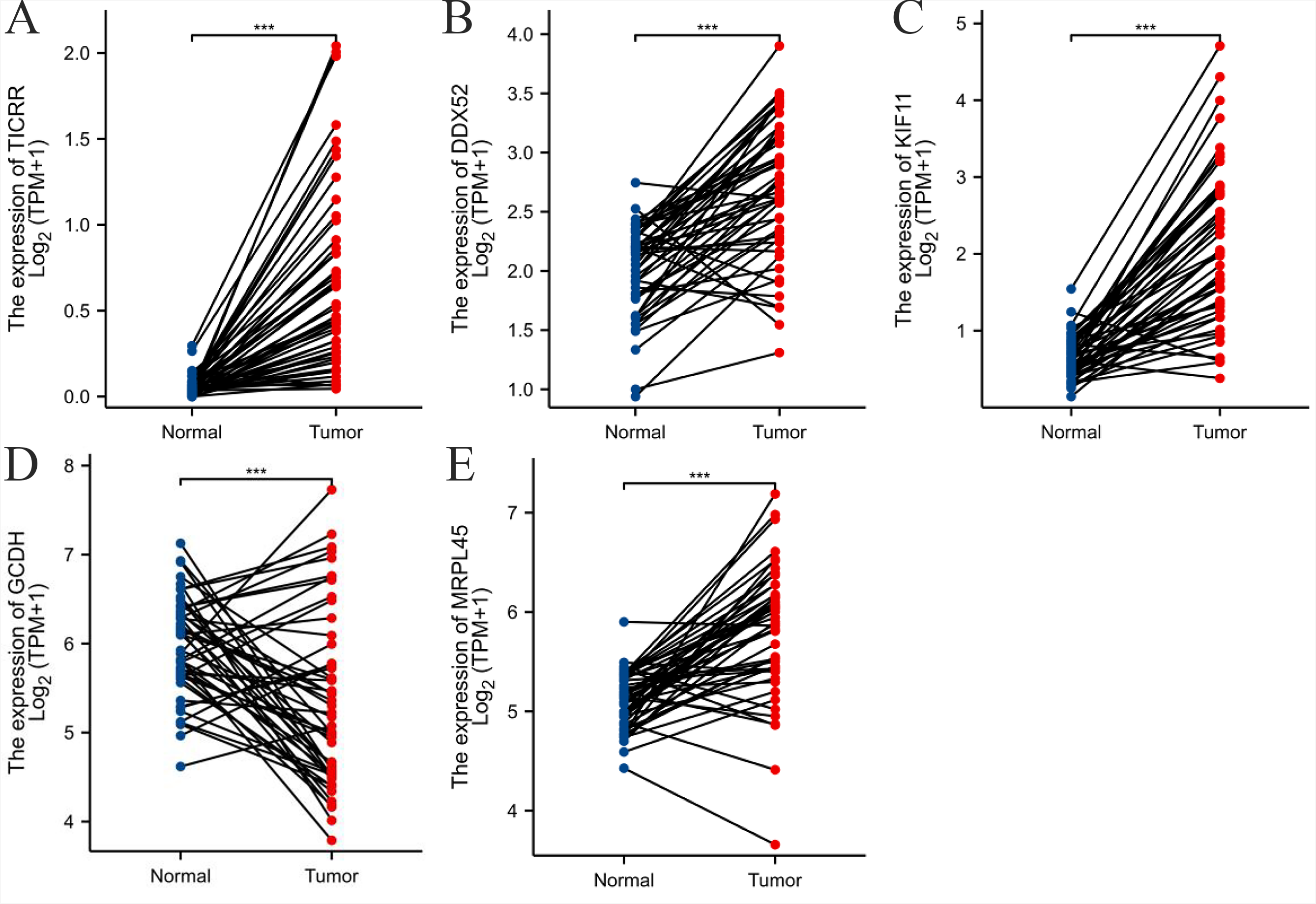

Supplement: Supplementary file 2 — Figure S4: Expression of TICRR, DDX52, KIF11, GCDH, and MRPL45 in the TCGA‐LIHC dataset. (A) The expression of TICRR in the TCGA‐LIHC dataset. (B) The expression of DDX52 in the TCGA‐LIHC dataset. (C) The expression of KIF11 in the TCGA‐LIHC dataset. (D) The expression of GCDH in the TCGA‐LIHC dataset. (E) The expression of MRPL45 in the TCGA‐LIHC dataset. [file FSN3-13-e71156-s001.tif]
